# Prescribing of Opioid Analgesics and Buprenorphine for Opioid Use Disorder During the COVID-19 Pandemic

**DOI:** 10.1001/jamanetworkopen.2021.6147

**Published:** 2021-04-15

**Authors:** Janet M. Currie, Molly K. Schnell, Hannes Schwandt, Jonathan Zhang

**Affiliations:** 1 Center for Health and Wellbeing, Princeton University, Princeton, New Jersey; 2 National Bureau of Economic Research, Cambridge, Massachusetts; 3 Department of Economics, Northwestern University, Evanston, Illinois; 4 School of Education and Social Policy, Northwestern University, Evanston, Illinois

## Abstract

**Importance:**

The COVID-19 pandemic disrupted medical care, impacting prescribing of opioid analgesics and buprenorphine for opioid use disorder. Understanding these patterns can help address barriers to care.

**Objective:**

To evaluate how prescribing of opioid analgesics and buprenorphine for opioid use disorder changed throughout the COVID-19 pandemic among both new and existing patients.

**Design, Setting, and Participants:**

In this cross-sectional study, use of opioid analgesics and buprenorphine for opioid use disorder from March 18 to September 1, 2020, was projected using a national database of retail prescriptions from January 1, 2018, to March 3, 2020. Actual prescribing was compared with projected levels for all, existing, and new patients.

**Exposures:**

The data include prescriptions to patients independent of insurance status or type and cover 90% of retail prescriptions, 70% of mail-order prescriptions, and 70% of nursing home prescriptions.

**Main Outcomes and Measures:**

Prescriptions for opioid analgesics and buprenorphine for opioid use disorder. Outcomes included total number of prescriptions, total morphine milligram equivalents, mean morphine milligram equivalents per prescription, mean dispensed units per prescription, and number of patients filling prescriptions.

**Results:**

A total of 452 691 261 prescriptions for opioid analgesics and buprenorphine for opioid use disorder were analyzed for 90 420 353 patients (50 921 535 female patients [56%]; mean [SD] age, 49 [20] years). From March 18 to May 19, 2020, 1877 million total morphine milligram equivalents of opioid analgesics were prescribed weekly vs 1843 million projected, a ratio of 102% (95% prediction interval [PI], 94%-111%; *P* = .71). The weekly number of opioid-naive patients receiving opioids was 370 051 vs 564 929 projected, or 66% of projected (95% PI, 63%-68%; *P* < .001). Prescribing of buprenorphine was as projected for existing patients, while the number of new patients receiving buprenorphine weekly was 9865 vs 12 008 projected, or 82% (95% PI, 76%-88%; *P* < .001). From May 20 to September 1, 2020, opioid prescribing for new patients returned to 100% of projected (95% PI, 96%-104%; *P* = .95), while the number of new patients receiving buprenorphine weekly was 10 436 vs 11 613 projected, or 90% (95% PI, 83%-97%; *P* = .009).

**Conclusions and Relevance:**

In this cross-sectional study, existing patients receiving opioid analgesics and buprenorphine for opioid use disorder generally maintained access to these medications during the COVID-19 pandemic. Opioid prescriptions for opioid-naive patients decreased briefly and then rebounded, while initiation of buprenorphine remained at a low rate through August 2020. Reductions in treatment entry may be associated with increased overdose deaths.

## Key Points

**Question:**

How has prescribing of opioid analgesics and buprenorphine for opioid use disorder changed throughout the COVID-19 pandemic?

**Findings:**

This cross-sectional study analyzed prescriptions from 90 420 353 patients and found that from March 18 to May 19, 2020, total morphine milligram equivalents of opioid analgesics prescribed to existing patients followed prepandemic trends; prescriptions to opioid-naive patients were 34% below projected levels but rebounded by August 2020. Prescribing of buprenorphine for opioid use disorder followed prepandemic trends for existing patients, while prescriptions to new patients were 18% below projected levels, rebounding to 90% of projected levels by August 2020.

**Meaning:**

This study suggests that prescriptions for opioid analgesics and buprenorphine for opioid use disorder decreased among new, but not existing, patients during the COVID-19 pandemic.

## Teaser

This cross-sectional study used a national database of retail prescriptions to evaluate how prescribing of opioid analgesics and buprenorphine for opioid use disorder changed throughout the COVID-19 pandemic among both new and existing patients.

## Introduction

Many medical services have been disrupted because of the COVID-19 pandemic.^1^ Deaths due to opioid overdoses have also increased, which may be associated with reductions in access to care.^2,3^ Disruptions in care may have affected new and existing patients differently. Patients taking prescription opioids may have experienced supply reductions, with possible negative outcomes regarding pain control and switching to nonprescription opioids (illegal drugs such as heroin and illicit fentanyl). Opioid-naive patients may also have been less likely to receive opioid prescriptions. Reduced access to treatment of opioid use disorder (OUD), even among patients with life-threatening episodes, has been documented.^4^ However, emergency Medicaid expansions and greater use of telemedicine may have improved access to treatment.^5^ Hence, to our knowledge, how prescribing of opioid analgesics and buprenorphine for OUD changed during the pandemic is unclear.

This study examined how prescribing of opioid analgesics for pain management and buprenorphine for OUD evolved from January 1, 2018, to September 1, 2020, for new and existing patients. We hypothesized that potential new patients would have the greatest changes in prescribing.

## Methods

### Prescription and Patient Data

Prescription data were drawn from IQVIA’s LRx database for the United States. These data include 2 billion prescriptions per year, representing more than 90% of prescriptions filled at retail pharmacies, 70% of mail-order medications, and 70% of medications used in long-term care. Data were drawn from electronic records from pharmacies, payers, software companies, and transactional clearinghouses. The data included 452 691 261 opioid analgesic prescriptions (eAppendix 2 in the Supplement) and buprenorphine prescriptions for OUD for 90 420 353 unique patients (50 921 535 female patients [56%]; mean [SD] age, 49 [20] years) (eTable in the Supplement). Race/ethnicity is not recorded in the IQVIA LRx database. We followed the Strengthening the Reporting of Observational Studies in Epidemiology (STROBE) reporting guideline for cross-sectional studies. All personal health information was removed or encrypted by a proprietary, automated deidentification engine prior to being collected by IQVIA. This process has been certified as compliant with the Health Insurance Portability and Accountability Act (HIPAA) and exempt from review by Princeton University’s institutional review board because the data have no personal identifying information, so the research was determined not to involve human participants.

Four weekly measures for opioid analgesic prescriptions were constructed: (1) total number of prescriptions filled, (2) total milligrams of morphine equivalents (MMEs), (3) mean MMEs per prescription, and (4) mean number of dispensed units (eg, pills) per prescription. The 3 analogous weekly measures for buprenorphine for OUD were (1) total number of prescriptions filled, (2) total number of units prescribed, and (3) mean number of dispensed units per prescription.

Methadone is used for OUD treatment in clinics and not usually filled through retail pharmacies. Methadone is also used for pain. Similarly, naltrexone is prescribed for both OUD and alcohol use disorder. A total of 4 718 469 methadone prescriptions and 3 055 333 naltrexone prescriptions were excluded from the data. These prescriptions accounted for 15% of sample buprenorphine, naltrexone, and methadone prescriptions. A total of 1 961 844 buprenorphine prescriptions in formulations used mainly for pain were included with opioid analgesics and were excluded from buprenorphine prescriptions for OUD; these prescriptions represent 4.7% of buprenorphine prescriptions.

Weekly numbers of unique patients filling prescriptions were also calculated. Patients who had not filled a prescription for an opioid analgesic in the past 365 days were considered opioid naive. Similarly, patients who had not filled a prescription for buprenorphine for OUD in the past 365 days were considered new patients. Other patients were classified as existing patients. Patient-level measures began in 2019 because of the 365-day lookback.

### Statistical Analysis

Plots of the variables already discussed are presented in addition to analysis of whether prescriptions deviated from projected patterns given prescriptions in prior years. Prescriptions for opioid analgesics have been decreasing over time, and buprenorphine prescriptions for OUD were increasing before the COVID-19 pandemic. Prescriptions typically decrease in weeks with major holidays.

Linear regressions of outcomes in the prepandemic period (from January 1, 2018, to March 3, 2020) were estimated on separate indicators denoting each week of the year (51 indicators), a linear time trend in weeks from the sample start, and an indicator denoting whether a major holiday occurred during a given week (eAppendix 1 in the Supplement). Coefficient estimates from this regression model were then used to project prescription and patient numbers during the COVID-19 pandemic, and ratios of actual to predicted outcomes were constructed. *P* values and 95% prediction intervals (PIs) show whether actual outcomes were significantly different than projected.

Projected values were calculated for 2 periods to capture the initial impact of COVID-19 and the subsequent return to more normal activity levels. The first period was from March 18 to May 19, 2020, beginning with President Trump’s declaration of a national emergency concerning the novel coronavirus disease outbreak^6^ and ending in the first week after early April that the 7-day moving average number of deaths was below 1500 per day.^7^ The second period was from May 20 to September 1, 2020. All data analyses were conducted using R, version 4.0.2 (R Group for Statistical Computing). All *P* values were from 2-sided tests, and results were deemed statistically significant at *P* < .05.

## Results

**Figure 1.**
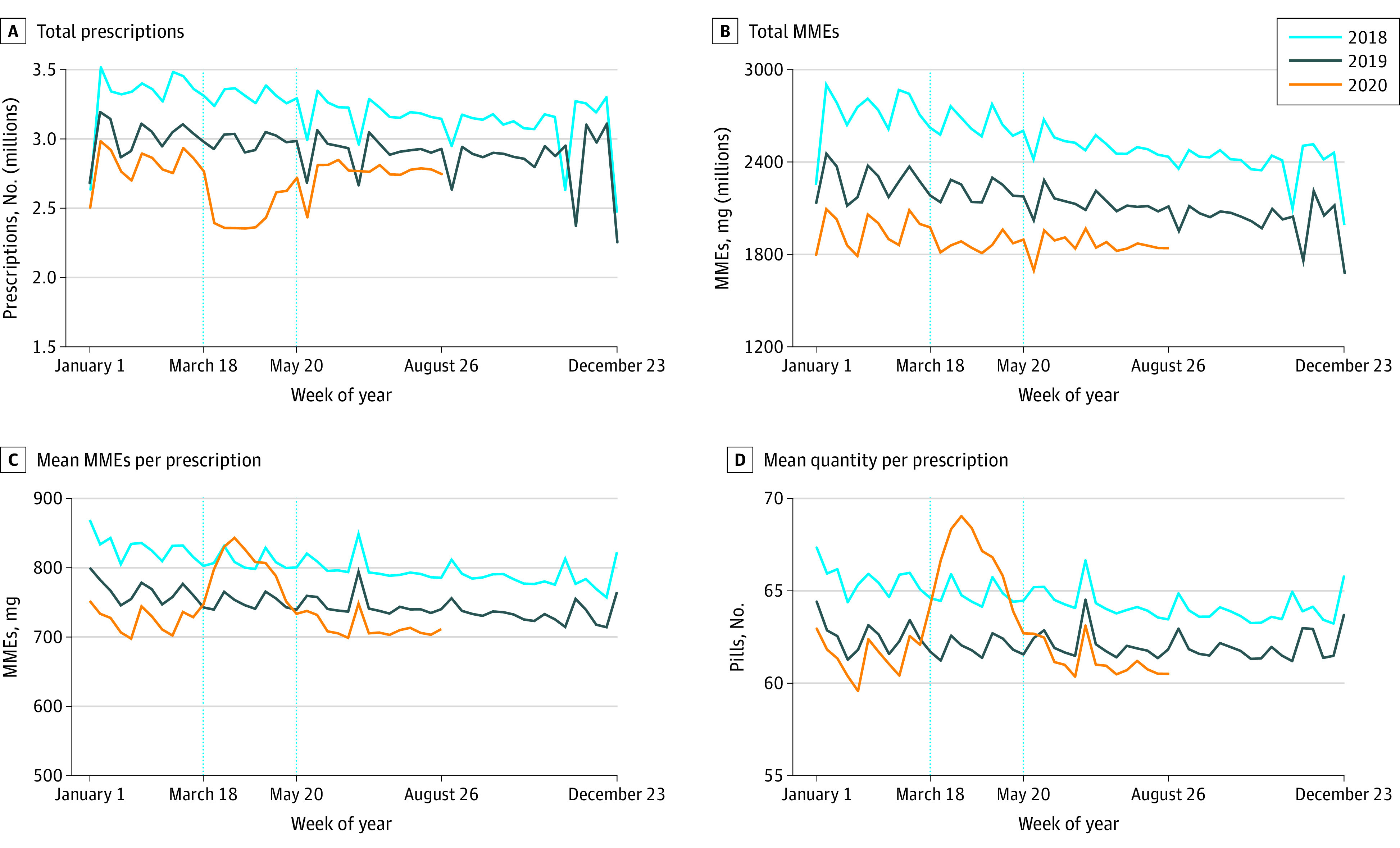
Weekly Prescription Measures for Opioid Analgesics A, Total weekly prescription counts. B, Total morphine milligram equivalents (MMEs). C, Mean MMEs per prescription. D, Mean number of dispensed units (eg, pills) per prescription. Each data point represents the measure over a 7-day period. Labels on the x-axes refer to the first day of the week. Dotted vertical lines correspond to the weeks starting on March 18 and May 20, 2020. These dates coincide with President Trump’s declaration of a national emergency concerning the novel coronavirus disease outbreak and the first week that the 7-day moving average number of deaths was below 1500 per day since early April 2020, respectively.

A total of 452 691 261 prescriptions for opioid analgesics and buprenorphine for OUD were analyzed for 90 420 353 patients (50 921 535 female patients [56%]; mean [SD] age, 49 [20] years). Figure 1 shows weekly measures for opioid analgesics in 2018, 2019, and 2020. Weekly opioid analgesic prescriptions decreased from March 18 to May 19, 2020, from a maximum of 2 733 260 prescriptions the first week to a minimum of 2 324 923 the week of April 15, 2020. Figure 1B shows, however, that total MMEs prescribed were flat, at 102% (1 877 047 372) of projected levels (1 843 349 191) in the first period (95% PI, 94%-111%; *P* = .71). Figure 1C and D shows that this is because mean MMEs (actual, 799; and projected, 698 [114%]; 95% PI, 112%-117%; *P* < .001) and the mean number of dispensed units per prescription increased (actual, 66.7; and projected, 60.0 [111%]; 95% PI, 109%-113%; *P* < .001). All these changes were short lived, with levels returning to prior levels after May 20, 2020.

**Figure 2.**
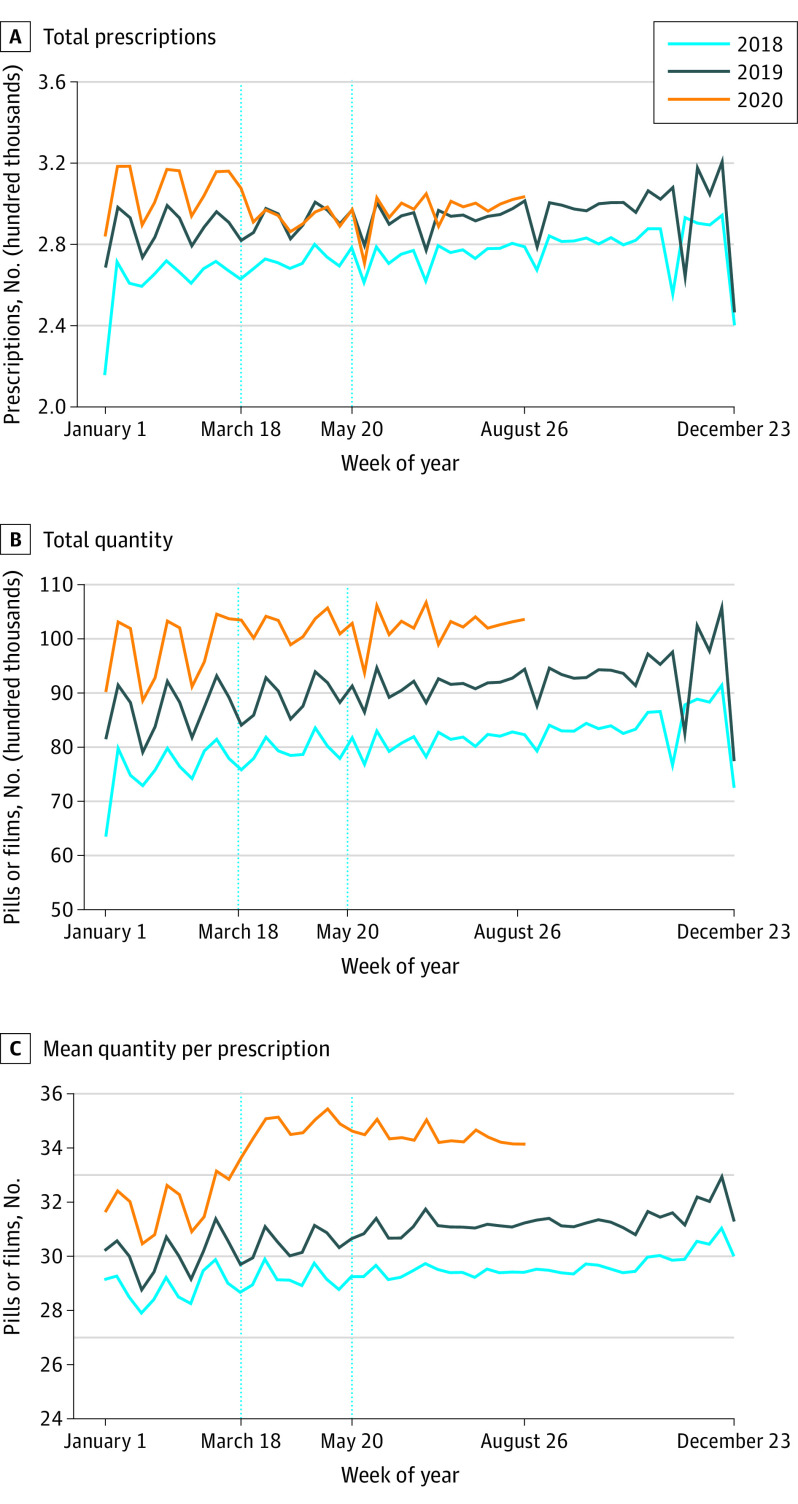
Weekly Prescription Measures for Buprenorphine for Opioid Use Disorder (OUD) A, Total weekly prescription counts. B, Total number of dispensed units (eg, pills). C, Mean number of dispensed units per prescription. Each data point represents the measure over a 7-day period. Labels on the x-axes refer to the first day of the week. Dotted vertical lines correspond to the weeks starting on March 18 and May 20, 2020. These dates coincide with President Trump’s declaration of a national emergency concerning the novel coronavirus disease outbreak and the first week that the 7-day moving average number of deaths was below 1500 per day since early April 2020, respectively.

Weekly prescription measures for buprenorphine for OUD are shown in Figure 2. Figure 2A shows a small decrease to 294 848 compared with 311 467 projected, or 95% (95% PI, 90%-100%; *P* = .04), in the number of prescriptions between March 18 and May 19, 2020. Figure 2B shows that the total number of units prescribed was flat in both periods. Stability in the overall quantity prescribed is due to an increase in the mean number of units per prescription to 35 compared with 32 projected, or 108% (95% PI, 106%-110%; *P* < .001) (Figure 2C).

**Figure 3.**
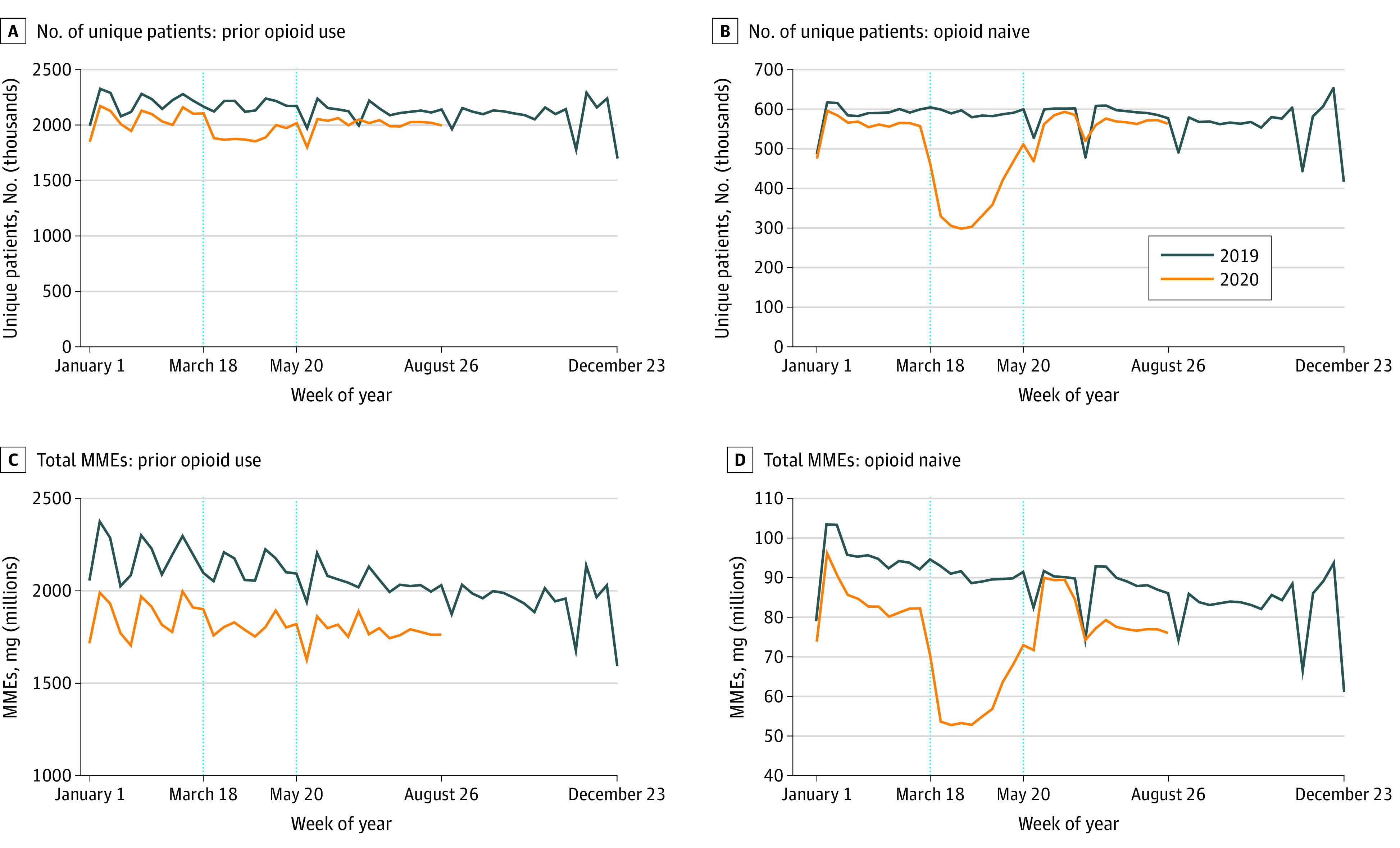
Weekly Number of Patients Filling Opioid Analgesic Prescriptions and Total Morphine Milligram Equivalents (MMEs) Filled: Existing vs New Patients Number of unique patients filling an opioid analgesic prescription in a given week (A and B) and total MMEs filled in a given week (C and D) for patients who previously received opioids (A and C) and opioid-naive patients (B and D). Patients are classified as opioid naive if they did not fill an opioid prescription in the past 365 days. Each data point represents the measure over a 7-day period. Labels on the x-axes refer to the first day of the week. Dotted vertical lines correspond to the weeks starting on March 18 and May 20, 2020. These dates coincide with President Trump’s declaration of a national emergency concerning the novel coronavirus disease outbreak and the first week that the 7-day moving average number of deaths was below 1500 per day since early April 2020, respectively.

Overall patterns in prescribing are dominated by the stock of existing patients who outnumber the inflow of new patients each period. Figure 3 distinguishes between new and existing patients receiving opioid analgesics. Only the comparison with 2019 is shown because of the 365-day lookback period to identify new patients. Figure 3 shows that the number of existing patients who filled an opioid prescription decreased slightly from March 18 to May 19, 2020, although Figure 3C shows that there was no change in total MMEs prescribed. In contrast, Figure 3B shows a decrease in the number of opioid-naive patients filling opioid analgesic prescriptions in the first period to 370 051 weekly compared with 564 929 projected, or 66% of projected levels (95% PI, 63%-68%; *P* < .001). This decrease was followed by a rebound to prior levels after May 2020 (558 293 new patients weekly compared with 559 040 projected, or 100% [95% PI, 96%-104%; *P* = .95]). Figure 3D shows that total MMEs prescribed to opioid-naive patients followed the same pattern.

**Figure 4.**
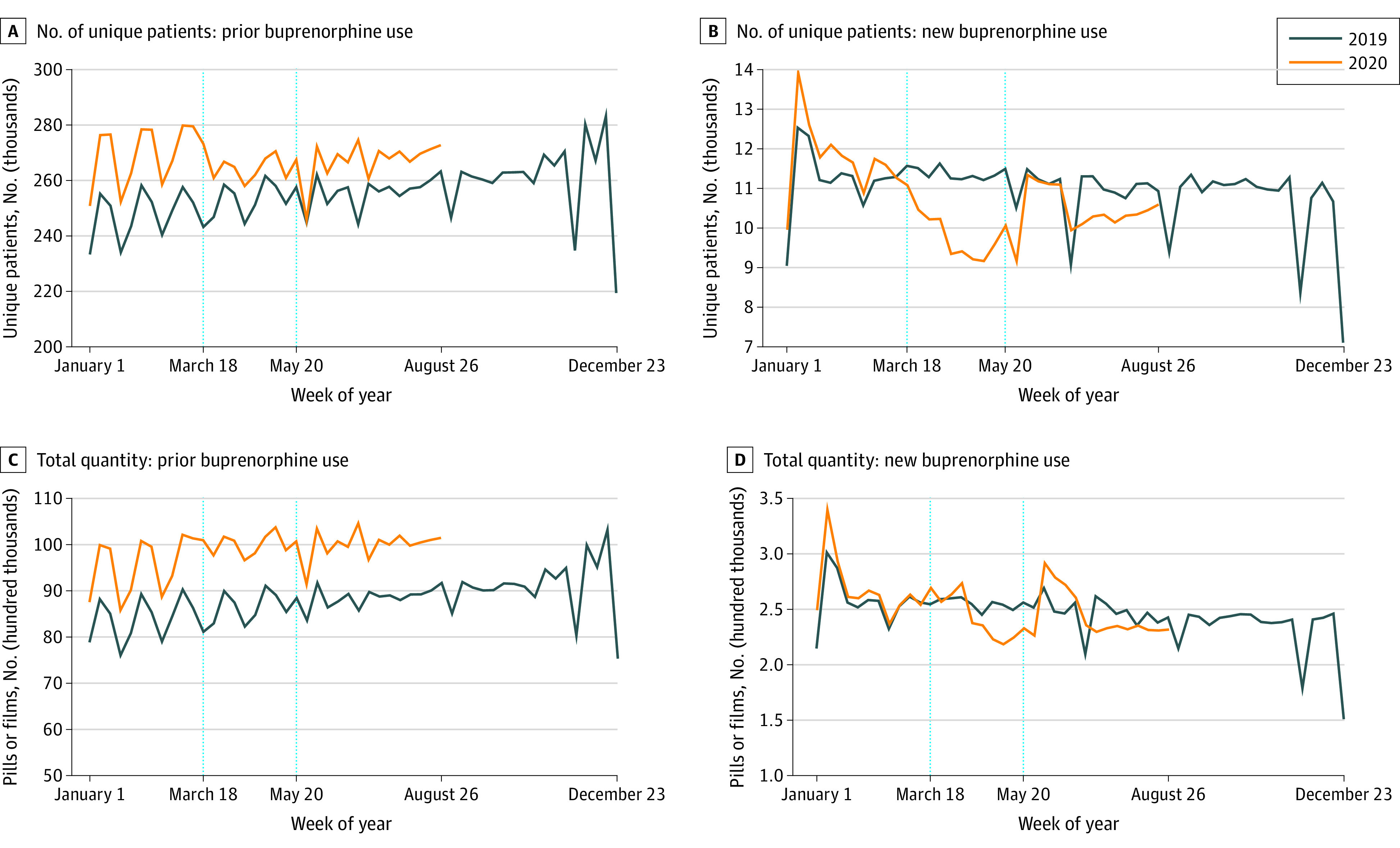
Weekly Number of Patients Filling Buprenorphine Prescriptions for Opioid Use Disorder (OUD) and Total Quantity Filled: Existing vs New Patients Number of unique patients filling a prescription for buprenorphine for OUD in a given week (A and B) and total number of dispensed units in a given week (C and D) for existing patients who previously received buprenorphine for OUD (A and C) and new patients who did not previously receive buprenorphine for OUD (B and D). Patients are classified as new if they did not fill a buprenorphine prescription for OUD in the past 365 days. Each data point represents the measure over a 7-day period. Labels on the x-axes refer to the first day of the week. Dotted vertical lines correspond to the weeks starting on March 18 and May 20, 2020. These dates coincide with President Trump’s declaration of a national emergency concerning the novel coronavirus disease outbreak and the first week that the 7-day moving average number of deaths was below 1500 per day since early April 2020, respectively.

Figure 4 shows buprenorphine prescriptions for OUD for new and existing patients. Figure 4A shows little change in the number of existing patients filling prescriptions; in the second period, for example, the number of existing patients filling prescriptions was 97% (267 427) of projected (276 535) levels (95% PI, 94%-100%; *P* = .03). Figure 4C indicates that there was no change in the total number of units of buprenorphine prescribed. Figure 4B indicates that in January and February 2020, patients entered buprenorphine treatment of OUD at higher levels than in early 2019. However, entry into buprenorphine treatment decreased from March 18 to May 19, 2020 (9865 each week compared with 12 008 projected, or 82% of projected levels [95% PI, 76%-88%; *P* < .001]), ending lower than in May 2019. Entry into treatment remained below 2019 levels through September 1, 2020.

The Table shows actual and projected measures for March 18 to May 19, 2020, and for May 20 to September 1, 2020. It also shows the ratio of actual to projected levels, 95% PIs, and *P* values indicating whether the actual levels were significantly different from the projected levels. Projected measures are based on the modeling already described. The models (available on request) have *R*^2^ values from 0.88 for total opioid analgesic prescriptions to 0.96 for number of new patients receiving buprenorphine for OUD.

**Table.** Projected and Actual Mean Weekly Counts of Prescription and Patient Measures From March 18 to May 19, 2020, and From May 20 to September 1, 2020^a^

Characteristic	March 18 to May 19, 2020				May 20 to September 1, 2020			
	Projected 2020 level (95% PI)	Actual 2020 level	Ratio of actual to projected 2020 level (95% PI)	*P* value	Projected 2020 level (95% PI)	Actual 2020 level	Ratio of actual to projected 2020 level (95% PI)	*P* value
**Weekly prescriptions**								
Opioid analgesics								
Prescriptions (thousands)	2752.15 (2544.21-2960.10)	2472.76	0.90 (0.84-0.97)	.007	2668.11 (2459.62-2876.60)	2753.09	1.03 (0.96-1.12)	.48
Total MMEs (millions)	1843.35 (1687.54-2000.16)	1877.05	1.02 (0.94-1.11)	.71	1743.14 (1585.91-1900.36)	1865.50	1.07 (0.98-1.18)	.19
MMEs per prescription	698.29 (680.98-715.59)	798.79	1.14 (1.12-1.17)	<.001	689.89 (672.54-707.24)	714.87	1.04 (1.00-1.06)	.008
Quantity per prescription	60.02 (58.82-61.22)	66.73	1.11 (1.09-1.13)	<.001	59.81 (58.61-61.02)	61.32	1.03 (1.01-1.05)	.02
Buprenorphine								
Prescriptions (thousands)	311.47 (295.21-327.72)	294.85	0.95 (0.90-1.00)	.04	315.19 (298.89-331.49)	297.55	0.94 (0.90-1.00)	.03
Total quantity (thousands)	9956.64 (9403.35-10 509.93)	10 241.06	1.03 (0.97-1.09)	.36	10 194.77 (9640.02-10 749.51)	10 243.86	1.00 (0.97-1.03)	.87
Quantity per prescription	32.08 (31.48-32.68)	34.73	1.08 (1.06-1.10)	<.001	32.53 (31.93-33.13)	34.43	1.06 (1.04-1.08)	<.001
**Weekly patients**								
Opioid analgesics								
Total No. of patients (thousands)								
Prior patients	2032.07 (1929.32-2134.82)	1924.16	0.95 (0.90-1.00)	.04	1977.76 (1875.01-2080.51)	2010.07	1.02 (0.97-1.07)	.57
Opioid naive	564.93 (543.12-586.74)	370.05	0.66 (0.63-0.68)	<.001	559.04 (537.23-580.85)	558.29	1.00 (0.96-1.04)	.95
Total MMEs (millions)								
Prior patients	1793.08 (1667.46-1918.70)	1818.50	1.01 (0.95-1.09)	.72	1717.03 (1591.41-1842.65)	1786.13	1.04 (0.97-1.12)	.34
Opioid naive	80.08 (73.22-86.94)	58.55	0.73 (0.67-0.80)	<.001	77.55 (70.69-84.40)	79.37	1.02 (0.94-1.12)	.64
Buprenorphine								
Total No. patients (thousands)								
Prior patients	272.92 (264.92-280.92)	265.19	0.97 (0.94-1.00)	.06	276.53 (268.54-284.53)	267.43	0.97 (0.94-1.00)	.03
New patients	12.01 (11.10-12.91)	9.86	0.82 (0.76-0.88)	<.001	11.61 (10.71-12.52)	10.44	0.90 (0.83-0.97)	.009
Total quantity (thousands)								
Prior patients	9684.91 (9173.37-10 196.45)	9995.32	1.03 (0.98-1.09)	.28	9941.33 (9429.79-10 452.88)	9999.47	1.01 (0.96-1.06)	.83
New patients	267.71 (233.75-301.68)	245.30	0.92 (0.81-1.05)	.21	260.88 (226.92-294.85)	244.39	0.94 (0.83-1.08)	.38

Abbreviations: MMEs, morphine milligram equivalents; OUD, opioid use disorder; PI, prediction interval.

^a^ Weekly prescription and patient measures for opioid analgesics and buprenorphine treatment of OUD during 2 periods: March 18 to May 19, 2020, and May 20 to September 1, 2020. March 18 is the week after President Trump’s declaration of a national emergency concerning the novel coronavirus disease outbreak, and May 20 is the first week that the 7-day moving average number of deaths was below 1500 per day since April 2020. For each period and outcome measure, projected 2020 values are constructed based on projections from a linear regression model using data from January 1, 2018, to March 3, 2020, controlling for week of the year, a linear time trend in weeks from the start of our sample, and an indicator denoting whether a major holiday occurred during a given week (eAppendix 1 in the Supplement). Actual 2020 measures and the ratio of actual to projected measures are also displayed. Two-sided 95% PIs are displayed for these ratios. The *P* values are for 2-sided *t* tests of whether the actual 2020 measure is equal to the projected value. See eFigure 1 in the Supplement for a visual representation of these results.

The first part of the Table shows results for opioid analgesics. In the first period, there were 2 472 759 weekly prescriptions compared with 2 752 154 projected, or 90% of projected levels (95% PI, 84%-97%; *P* = .007). Prescriptions were 103% (2 753 087) of projected levels (2 668 109) in the second period (95% PI, 96%-112%; *P* = .48). In contrast, total MMEs were 102% (1 877 047 372) of projected levels (1 843 349 191) in the first period (95% PI, 94%-111%; *P* = .71), indicating no significant deviation from projected levels. Similarly, there was no significant deviation from projected MMEs in the second period. The decrease in the total number of prescriptions in the first period was offset by a statistically significant increase in mean MMEs (actual, 799; and projected, 698 [114%]; 95% PI, 112%-117%; *P* < .001) and in the mean number of dispensed units per prescription (actual, 66.7; and projected, 60.0 [111%]; 95% PI, 109%-113%; *P* < .001). Increases in the mean size of prescriptions continued into the second period, although the increases relative to the projected levels were smaller than in the first period.

The next part of the Table shows patterns for buprenorphine treatment of OUD. In the first period, weekly prescriptions decreased to 294 848 compared with 311 467 projected, or 95% (95% PI, 90%-100%; *P* = .04). Buprenorphine prescriptions for OUD were 94% (297 546) of projected levels (315 189) in the second period (95% PI, 90%-100%; *P* = .03). The overall quantity prescribed was not significantly affected given significant increases in the mean number of dispensed units per prescription. In the first period, the number of units increased to 35 compared with 32 projected, or 108% (95% PI, 106%-110%; *P* < .001). The increase in the second period was similar.

Distinguishing between existing patients and opioid-naive patients, the Table shows a small decrease in the number of existing patients filling opioid analgesic prescriptions in the first period. There was no change in total MMEs prescribed for existing patients in the first or second periods. Among opioid-naive patients, the number filling opioid analgesic prescriptions in the first period decreased to 370 051 weekly compared with 564 929 projected, or 66% of projected levels (95% PI, 63%-68%; *P* < .001). In the second period, there were 558 293 new patients weekly compared with 559 040 projected, or 100% (95% PI, 96%-104%; *P* = .95).

The final section of the Table distinguishes between new and existing patients receiving buprenorphine for OUD. Among existing patients, there were only slight decreases in the number of prescriptions filled in both the first and second periods. In the second period, for example, the number of existing patients filling prescriptions was 97% (267 427) of projected (276 535) levels (95% PI, 94%-100%; *P* = .03). There was no change in the total number of units of buprenorphine prescribed to existing patients in either period (first period: actual, 103% [9 995 319] of projected [9 684 911] [95% PI, 98%-109%]; *P* = .28; second period: actual, 101% [9 999 472] of projected [9 941 332] [95% PI, 96%-106%]; *P* = .83).

In contrast, the number of new patients receiving buprenorphine for OUD in the first period was 9865 each week compared with 12 008 projected, or 82% of projected levels (95% PI, 76%-88%; *P* < .001). In the second period, the number of new patients entering treatment was 90% (10 436) of projected (11 613) levels (95% PI, 83%-97%; *P* = .009).

## Discussion

Existing patients taking opioid analgesics experienced little disruption in their supply of such medications during the COVID-19 pandemic. Although they initially received fewer prescriptions, each prescription was for a larger quantity. Therefore, there was little change in total MMEs supplied. This finding also holds within each census region (eFigure 2 in the Supplement).

In contrast, opioid-naive patients were much less likely to be prescribed opioids from mid-March to May 2020, although prescribing quickly returned to projected levels. Estimates imply that from March 18 to May 19, 2020, 1.75 million fewer opioid-naive patients received opioid prescriptions than projected ([1 − 0.6550] × 564 929 patients × 9 weeks). To the extent that prescribing was permanently deferred or replaced with nonnarcotic analgesic medications, this pause in opioid prescribing may be associated with reductions in future opioid use in the affected cohort.^8^

Existing patients were also largely able to maintain access to buprenorphine for OUD throughout the pandemic. Although slightly fewer existing patients filled prescriptions each week, the prescriptions that were filled were larger, on average.

The biggest and most persistent changes in prescribing over the pandemic were experienced by new patients who might otherwise have received buprenorphine for OUD. Prescribing of buprenorphine for OUD to new patients decreased relative to projected levels and remained depressed through August 2020. This decrease was most pronounced in the Northeast (eFigure 3 in the Supplement). Estimates suggest that 36 954 additional patients with OUD would have received treatment in the absence of the pandemic ([(1 − 0.8215) × 12 008 patients × 9 weeks] + [(1 − 0.8986) × 11 613 patients × 15 weeks]). This reduction in treatment may have been associated with the increase in overdose deaths during the pandemic.

Increased reliance on telemedicine facilitated by changes in federal regulations^9^ may have helped existing patients maintain access to medications. HIPAA penalties were waived for good faith use of telehealth, and the number of days of take-home medications allowed was liberalized. The Drug Enforcement Administration waived the Ryan-Haight Act, which allowed teleprescribing of controlled substances without an initial in-person visit. Nevertheless, there were decreases in the number of new patients entering buprenorphine treatment of OUD relative to projected levels. It may be easier to engage existing patients via telemedicine than to serve new ones.^10^ Large pandemic-related reductions in visits to the emergency department^11^ may also have reduced the rate of initiation of new buprenorphine treatment of OUD.

### Strengths and Limitations

Strengths of the analysis stem from the comprehensive nature of the data and their timeliness. This study also has some limitations; one is that we cannot identify medications for OUD that do not flow through retail pharmacies, and we are also unable to assess whether some medications are being prescribed for alcohol use disorder rather than for OUD. Because of these considerations, methadone and naltrexone were excluded from the analysis. A second limitation is that it is possible that some prescriptions that were filled were not consumed. Finally, we cannot examine the association between initiation of buprenorphine treatment of OUD and overdose prevention.

## Conclusions

The results suggest that existing patients taking opioid analgesics experienced little disruption in access during the COVID-19 pandemic. Clinicians adjusted the size of prescriptions to compensate for reductions in the number of prescriptions filled. On the other hand, estimates imply that 1.75 million fewer opioid-naive patients received opioid prescriptions in the weeks from March 18 to May 19, 2020. Given the decreasing trends in opioid prescribing before the pandemic, some of these patients may never receive opioids, thereby reducing future use.

Existing patients likewise maintained access to buprenorphine treatment of OUD. However, the number of new patients receiving buprenorphine for OUD decreased by almost one-fourth at the beginning of the pandemic and had returned to only 90% of projected levels by the end of August. Estimates imply that 36 954 fewer patients entered treatment, which may have been associated with increases in overdose deaths during the pandemic.

These results suggest that the pandemic reversed some of the improvement in access to buprenorphine for OUD that occurred during the past decade.^12^ Improving and maintaining access to treatment should be a priority.

## Notes

Supplement. **eAppendix 1.** Prediction Equation **eTable.** Opioid Analgesics and Buprenorphine Prescriptions by Patient Age and Sex **eAppendix 2.** List of Included Opioid Analgesics **eFigure 1.** Forest Plot of Results from the Table **eFigure 2.** Total Morphine Milligram Equivalents by Census Region **eFigure 3.** New Patients Receiving Buprenorphine for OUD by Census Region
