# Supplementary material for: Prescribing of Opioid Analgesics and Buprenorphine for Opioid Use Disorder During the COVID-19 Pandemic
Source: JAMA Netw Open. 2021 Apr 15;4(4):e216147. doi: 10.1001/jamanetworkopen.2021.6147 (PMC8050741; doi:10.1001/jamanetworkopen.2021.6147)
Supplement: Supplement. — eAppendix 1. Prediction Equation eTable. Opioid Analgesics and Buprenorphine Prescriptions by Patient Age and Sex eAppendix 2. List of Included Opioid Analgesics eFigure 1. Forest Plot of Results from the Table eFigure 2. Total Morphine Milligram Equivalents by Census Region eFigure 3. New Patients Receiving Buprenorphine for OUD by Census Region [file jamanetwopen-e216147-s001.pdf]

Supplementary Online Content

Currie JM, Schnell MK, Schwandt H, Zhang J. Prescribing of opioid analgesics and buprenorphine for opioid use disorder during the COVID-19 pandemic. *JAMA Netw Open.* 2021;4(4):e216147. doi:10.1001/jamanetworkopen.2021.6147

eAppendix 1. Prediction Equation
eTable. Opioid Analgesics and Buprenorphine Prescriptions by Patient Age and Sex
eAppendix 2. List of Included Opioid Analgesics
eFigure 1. Forest Plot of Results from the Table
eFigure 2. Total Morphine Milligram Equivalents by Census Region
eFigure 3. New Patients Receiving Buprenorphine for OUD by Census Region

This supplementary material has been provided by the authors to give readers additional information about their work.

© 2021 Currie JM et al. *JAMA Network Open.*

eAppendix 1. Prediction Equation

We use the following equation to estimate predicted values:

$$Y_w = \beta \times w + \sum_{k=1}^{52} \alpha_k \times 1\{CalendarWeek_w = k\} + \mu \times Holiday_w + \epsilon_w$$

where Y_w denotes either a prescription outcome (e.g., weekly count of total opioid analgesic prescriptions) or a patient outcome (e.g., weekly count of new buprenorphine users), $\beta \times w$ is a linear time-trend starting in the first week of 2018, $1\{CalendarWeek_w = k\}$ is a full set of indicator variables for each week in the calendar year, $Holiday_w$ is an indicator variable denoting whether a holiday falls on week w, and ϵ_w is an error term. We estimate this equation using data from before the onset of the pandemic in the United States (January 1, 2018 to March 3, 2020).

© 2021 Currie JM et al. *JAMA Network Open.*

eTable. Opioid Analgesics and Buprenorphine Prescriptions by Patient Age and Sex

	Analgesic or Buprenorphine	Opioid Analgesics	Buprenorphine
% Female	0.56	0.57	0.44
% Male	0.43	0.43	0.55
Average Age	49.0	49.2	40.7
Age: % 0-24	0.13	0.13	0.06
Age: % 25-44	0.29	0.28	0.61
Age: % 45-64	0.33	0.34	0.27
Age: % 65+	0.25	0.25	0.06

Notes: The IQVIA LRx data include information on patient gender and age. This table presents a breakdown of prescriptions for opioid analgesics and buprenorphine along these dimensions. The IQVIA LRx data unfortunately do not include information on patient race.

© 2021 Currie JM et al. *JAMA Network Open.*

eAppendix 2. List of Included Opioid Analgesics

The opioid analgesic data include all pure and combination products involving the following active ingredients: butorphanol, codeine, dihydrocodeine, fentanyl, hydrocodone, hydromorphone, levorphanol, meperidine, morphine, nalbuphine, opium, oxycodone, oxymorphone, pentazocine, tapentadol, and tramadol.

In addition, we use a flag provided by IQVIA to identify formulations of buprenorphine that are used primarily for the treatment of pain rather than for the treatment of opioid use disorder (OUD). IQVIA classifies these formulations using National Drug Codes. We include buprenorphine formulations that are predominately used for pain management in our measures of opioid analgesics. There were 1,961,844 such prescriptions of buprenorphine in our data; these represent 4.7% of total buprenorphine prescriptions that we observe.

© 2021 Currie JM et al. *JAMA Network Open.*

eFigure 1. Forest Plot of Results from the Table

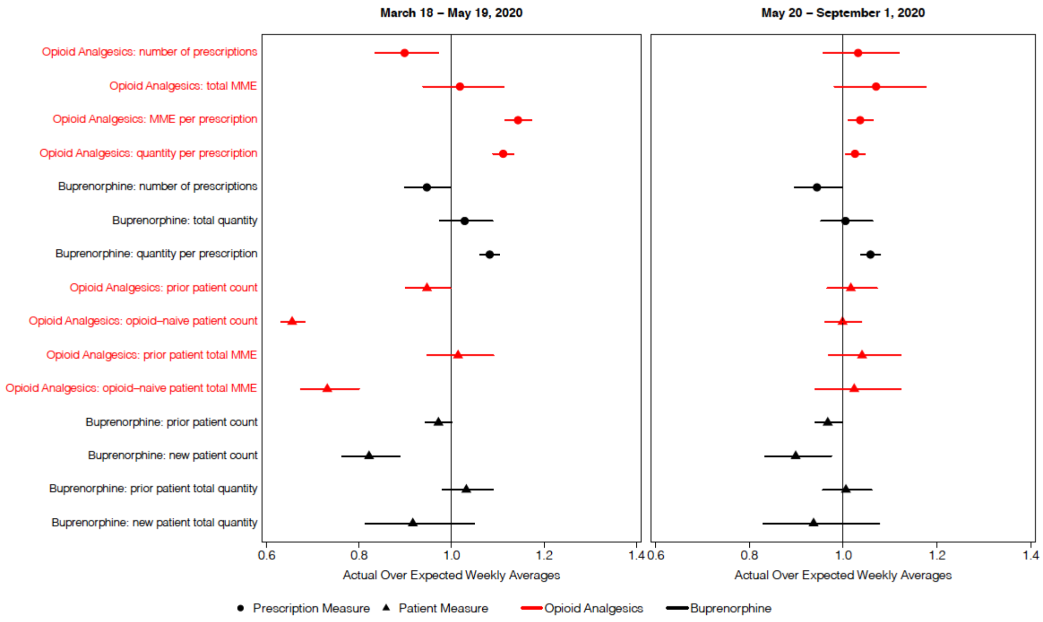

Notes: This figure provides a visual representation of the results shown in the Table in the main paper.

© 2021 Currie JM et al. *JAMA Network Open.*

eFigure 2. Total Morphine Milligram Equivalents (MMEs) by Census Region

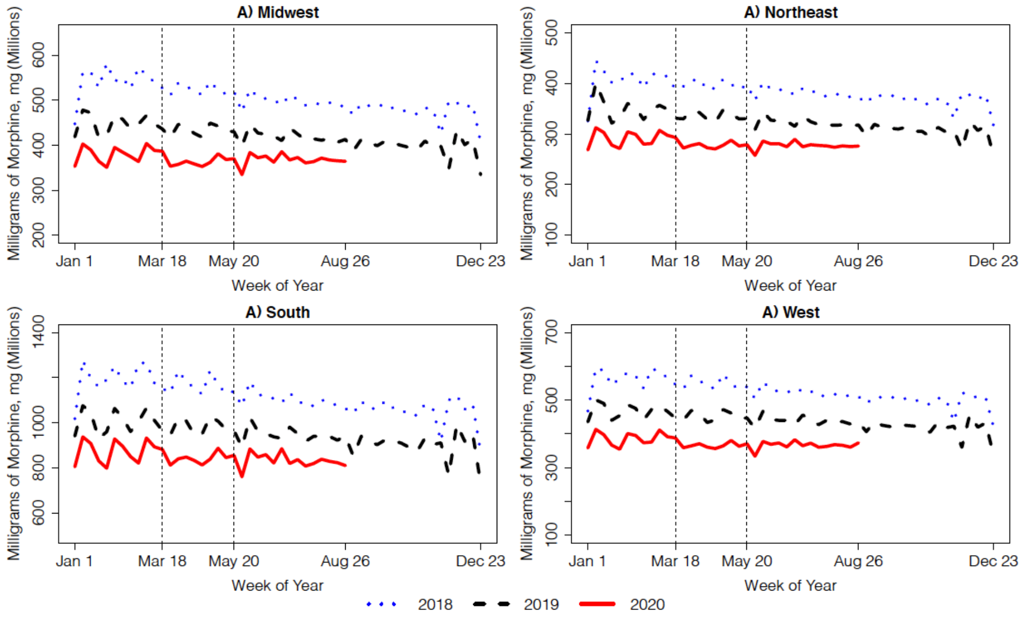

Note: This figure corresponds to Figure 1B in the main text. Prescriptions are assigned to census regions based on the provider's ZIP code on the script. New patient status is defined with respect to the first prescription ever in our data for that patient (in the entire country) and is not specific to census region. The figure shows that total MMEs of opioid analgesics prescribed remained constant throughout the pandemic in all four census regions.

© 2021 Currie JM et al. *JAMA Network Open.*

eFigure 3. New Patients Receiving Buprenorphine for OUD by Census Region

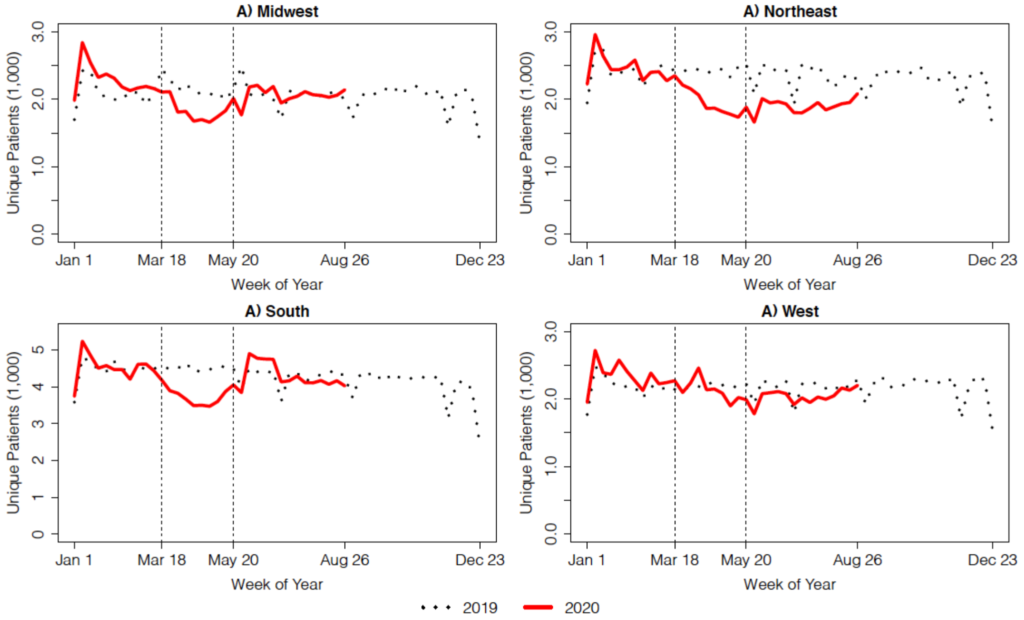

Notes: This figure corresponds to Figure 4B in the main text. The figure shows that prescriptions of buprenorphine for the treatment of OUD declined early in the pandemic, with the largest drop in the Northeast and the smallest drop in the West. Prescriptions returned to previous levels in the South and Midwest but remained depressed in the Northeast and (to some extent) in the West.

© 2021 Currie JM et al. *JAMA Network Open.*
